# Supplementary material for: Graph-structured populations elucidate the role of deleterious mutations in long-term evolution
Source: Nat Commun. 2025 Mar 10;16:2355. doi: 10.1038/s41467-025-57552-9 (PMC11894086; doi:10.1038/s41467-025-57552-9)
Supplement: Supplementary file 1 — Supplementary Information [file 41467_2025_57552_MOESM1_ESM.pdf]

Supplementary Information for Graph-structured populations elucidate the role of deleterious mutations in long-term evolution

Nikhil Sharma, Suman G. Das, Joachim Krug, and Arne Traulsen

CONTENTS

Supplementary Note 1: Exact formula for the fixation probability of a mutant on the star graph under dB and Bd updating 1

Supplementary Note 2: Probabilistic moving of offspring – The ω-process 4

Supplementary Note 3: Matrix approach to compute fixation probability on a random graph 5

1. Transition matrix for the Bd process 6
2. Transition matrix for dB process 7

Supplementary Note 4: Long-term evolution on regular graphs 7

Supplementary Note 5: Reversibility 10

Supplementary Note 6: Standard deviation in the steady-state fitness distribution 13

Supplementary Note 7: Long-term evolution on discrete fitness space 15

Supplementary Note 8: Criterion for a graph to have higher steady-state fitness than the complete graph 15

Supplementary Note 9: Effects of deleterious mutants on the initial phase of the long-term dynamics 17

Supplementary Note 10: Amplifier of fixation and suppressor of fixation in a metapopulation model 18

References 20

SUPPLEMENTARY NOTE 1: EXACT FORMULA FOR THE FIXATION PROBABILITY OF A MUTANT ON THE STAR GRAPH UNDER DB AND BD UPDATING

The state of the population can be described by $(\bullet/\circ, i)$ where the first index indicates if the central node is occupied by a mutant $(\bullet)$ or not $(\circ)$ and the second index gives the number of mutants in the leaf nodes. Let us denote $\phi_i^\bullet$ as the fixation probability of the mutant type when started with i mutant individuals in the leaf nodes and a mutant at the central node. Similarly, ϕ_i° is the fixation probability of the mutant type when started

with i mutant individuals in the leaf nodes with the central node occupied by a wild-type individual. With n number of leaves, $\phi_i^\bullet$ and ϕ_i° satisfy the following recursion relations [1],

$$\begin{aligned}
\phi_i^\bullet &= T_{i,i+1}^{\bullet\bullet}\phi_{i+1}^\bullet + T_{i,i}^{\bullet\circ}\phi_i^\circ + (1 - T_{i,i+1}^{\bullet\bullet} - T_{i,i}^{\bullet\circ})\phi_i^\bullet, \quad 0 \le i \le n-1, \\
\phi_i^\circ &= T_{i,i-1}^{\circ\circ}\phi_{i-1}^\circ + T_{i,i}^{\circ\bullet}\phi_i^\bullet + (1 - T_{i,i+1}^{\circ\circ} - T_{i,i}^{\circ\bullet})\phi_i^\circ, \quad 1 \le i \le n,
\end{aligned} \tag{1}$$

where

- $T_{i,i+1}^{\bullet\bullet}$ is the probability to transition from the state $(\bullet, i)$ to the state $(\bullet, i+1)$.
- $T_{i,i}^{\bullet\circ}$ is the probability to transition from the state $(\bullet, i)$ to the state $(\circ, i)$.
- $T_{i,i-1}^{\circ\circ}$ is the probability to transition from the state $(\circ, i)$ to the state $(\circ, i-1)$.
- $T_{i,i}^{\circ\bullet}$ is the probability to transition from the state $(\circ, i)$ to the state $(\bullet, i)$.

On rearranging the recursion relations we get,

$$\begin{aligned}
\phi_i^\bullet &= \pi_{i,i+1}^{\bullet\bullet}\phi_{i+1}^\bullet + \pi_{i,i}^{\bullet\circ}\phi_i^\circ, \quad 0 \le i \le n, \\
\phi_i^\circ &= \pi_{i,i-1}^{\circ\circ}\phi_{i-1}^\circ + \pi_{i,i}^{\circ\bullet}\phi_i^\bullet, \quad 1 \le i \le n,
\end{aligned} \tag{2}$$

where,

- $\pi_{i,i+1}^{\bullet\bullet}$ is the conditional probability to transition from the state $(\bullet, i)$ to the state $(\bullet, i+1)$ given that the number of mutants changes.
- $\pi_{i,i}^{\bullet\circ}$ is the conditional probability to transition from the state $(\bullet, i)$ to the state $(\circ, i)$ given that the number of mutants changes.
- $\pi_{i,i-1}^{\circ\circ}$ is the conditional probability to transition from the state $(\circ, i)$ to the state $(\circ, i-1)$ given that the number of mutants changes.
- $\pi_{i,i}^{\circ\bullet}$ is the conditional probability to transition from the state $(\circ, i)$ to the state $(\bullet, i)$ given that the number of mutants changes.

The conditional probabilities are given by,

$$\begin{aligned}
\pi_{i,i+1}^{\bullet\bullet} &= \frac{T_{i,i+1}^{\bullet\bullet}}{T_{i,i+1}^{\bullet\bullet} + T_{i,i}^{\bullet\circ}} = 1 - \pi_{i,i}^{\bullet\circ}, \\
\pi_{i,i-1}^{\circ\circ} &= \frac{T_{i,i-1}^{\circ\circ}}{T_{i,i-1}^{\circ\circ} + T_{i,i}^{\circ\bullet}} = 1 - \pi_{i,i}^{\circ\bullet},
\end{aligned} \tag{3}$$

For the Moran dB updating the transition probabilities are,

$$T_{i,i+1}^{\bullet\bullet} = \frac{n-i}{n+1} \quad \text{and} \quad T_{i,i}^{\bullet\circ} = \frac{1}{n+1}\frac{n-i}{ir+n-i}. \tag{4}$$

$$T_{i,i-1}^{\circ\circ} = \frac{i}{n+1} \quad \text{and} \quad T_{i,i}^{\circ\bullet} = \frac{1}{n+1}\frac{ir}{ir+n-i}. \tag{5}$$

Consequently the conditional probabilities are,

$$\pi^{\bullet\bullet}_{i,i+1} = \frac{n-i+ir}{n-i+ir+1} \quad \text{and} \quad \pi^{\circ\circ}_{i,i-1} = \frac{ir+n-i}{ir+n-i+r}. \tag{6}$$

From the ref. [2], we know that the probability of fixation of a mutant appearing at the center node is

$$\phi^{\bullet}_{\mathrm{dB},\bigstar}(f',f) = \frac{\pi^{\bullet\bullet}_{0,1}}{A(1,n)} \tag{7}$$

where,

$$A(l,m) = 1 + \sum_{j=l}^{m-1} \pi^{\bullet\circ}_{j,j} \prod_{k=l}^{j} \frac{\pi^{\circ\circ}_{k,k-1}}{\pi^{\bullet\bullet}_{k,k+1}}. \tag{8}$$

After substituting for the conditional probabilities, we get

$$\phi^{\bullet}_{\mathrm{dB},\bigstar}(f',f) = \frac{N-1}{N}\frac{1}{1+\frac{N-2}{1+(N-1)\frac{f'}{f}}}. \tag{9}$$

Similarly, we find

$$\phi^{\circ}_{\mathrm{dB},\bigstar}(f',f) = \frac{\pi^{\circ\bullet}_{1,1}}{A(1,n)} = \frac{\frac{f'}{f}}{\left(N-2+2\frac{f'}{f}\right)\left(1+\frac{N}{1+(N-1)\frac{f'}{f}}\right)}. \tag{10}$$

$\phi^{\bullet}_{\mathrm{dB},\bigstar}$ and $\phi^{\circ}_{\mathrm{dB},\bigstar}$ are used to compute uniform and temperature initialised dB fixation probabilities for the star graph.

The uniform (when offspring move to vacant node) and temperature (when parent move to vacant node) initialised fixation probability under Moran dB updating are,

$$\begin{aligned}\Phi^{\mathcal{U}}_{\mathrm{dB},\bigstar}(f',f) = \Phi_{\mathrm{dB}^o,\bigstar}(f',f) &= \frac{\phi^{\bullet}_{\mathrm{dB},\bigstar}(f',f)+(N-1)\phi^{\circ}_{\mathrm{dB},\bigstar}(f',f)}{N},\\ \Phi^{\mathcal{T}}_{\mathrm{dB},\bigstar}(f',f) = \Phi_{\mathrm{dB}^p,\bigstar}(f',f) &= \frac{(N-1)\phi^{\bullet}_{\mathrm{dB},\bigstar}(f',f)+\phi^{\circ}_{\mathrm{dB},\bigstar}(f',f)}{N},\end{aligned} \tag{11}$$

From Fig. 2B (main text), the uniform initialised star graph is a suppressor of selection [3, 4] whereas the temperature initialised star graph is an amplifier of fixation.

Similarly, for Bd updating,

$$\phi^{\bullet}_{\mathrm{Bd},\bigstar}(f',f) = \frac{\left(\left(\frac{f'}{f}\right)^2-1\right)\left(N-1+\frac{f'}{f}\right)}{\frac{f'}{f}\left(\frac{f'}{f}\left(N-1+\frac{f'}{f}\right)-\left((N-1)\frac{f'}{f}+1\right)\left(\frac{N-1+\frac{f'}{f}}{(N-1)\left(\frac{f'}{f}\right)^2+\frac{f'}{f}}\right)^{N-1}\right)}, \tag{12}$$

and the fixation probability for a mutant initially placed on a leaf node is,

$$\phi^{\circ}_{\mathrm{Bd},\bigstar}(f',f) = \frac{(N-1)\left(\left(\frac{f'}{f}\right)^2-1\right)\left(N-1+\frac{f'}{f}\right)}{\left((N-1)\frac{f'}{f}+1\right)\left(\frac{f'}{f}\left(N-1+\frac{f'}{f}\right)+\left((N-1)\frac{f'}{f}+1\right)\left(\frac{N-1+\frac{f'}{f}}{(N-1)\left(\frac{f'}{f}\right)^2+\frac{f'}{f}}\right)^{N-1}\right)}. \tag{13}$$

Eqs. 12, 13 are used to compute uniform (parent moving) and temperature (offspring moving) initialised fixation probabilities for the star graph under Bd updating,

$$\begin{aligned}\Phi^{\mathcal{U}}_{\mathrm{Bd},\bigstar}(f',f) = \Phi_{\mathrm{Bd}^p,\bigstar}(f',f) &= \frac{\phi^{\bullet}_{\mathrm{Bd},\bigstar}(f',f)+(N-1)\phi^{\circ}_{\mathrm{Bd},\bigstar}(f',f)}{N},\\ \Phi^{\mathcal{T}}_{\mathrm{Bd},\bigstar}(f',f) = \Phi_{\mathrm{Bd}^o,\bigstar}(f',f) &= \frac{(N-1)\phi^{\bullet}_{\mathrm{Bd},\bigstar}(f',f)+\phi^{\circ}_{\mathrm{Bd},\bigstar}(f',f)}{N},\end{aligned} \tag{14}$$

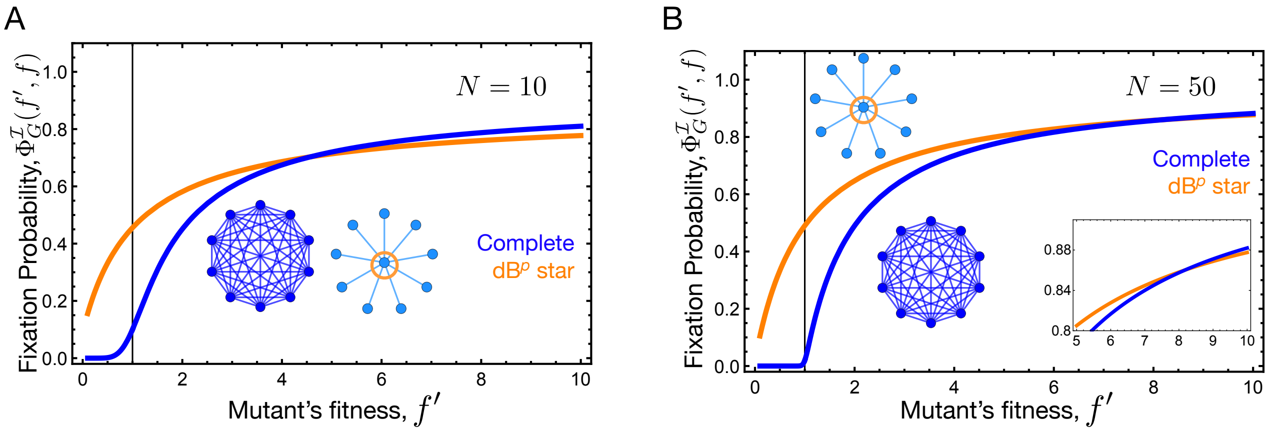

FIG. 1. **The star graph, a piecewise amplifier of fixation for finite N.** Panel A, the star graph under dB^p updating is a piecewise amplifier of fixation for finite population size N. For finite N, the probability of fixation of a mutant with fitness f' on the star graph is higher than the complete graph for $f' \leq f^*$ and lower for $f' > f^*$. From panel B, we can see that the value of threshold fitness f^* increases on increasing the population size. Only in the limit of infinite population size, the star graph becomes a true amplifier of fixation for dB^p updating.

SUPPLEMENTARY NOTE 2: PROBABILISTIC MOVING OF OFFSPRING – THE ω-PROCESS

So far we have looked into the fixation probabilities for either the case of offspring moving or the parent moving. We now investigate the fixation probability on the star graph when with probability ω offsprings move and with probability $1-\omega$ parents move. For rare mutations, the choice of individual moving only affects the mutant initialisation distribution. Once a mutant is initialised in a monomorphic resident population, the subsequent

fixation/extinction dynamics remains unaffected by the type of individual moving to vacant sites. Therefore, the ω offspring-parent process for a given birth-death update rule is the convex combination of the corresponding uniform and temperature initialised fixation probabilities. This way, the ω fixation probability for the dB updating is,

$$\Phi_{\mathrm{dB},\bigstar}(f',f) = \omega \cdot \Phi^{\mathcal{U}}_{\mathrm{dB},\bigstar}(f',f) + (1-\omega)\cdot \Phi^{\mathcal{T}}_{\mathrm{dB},\bigstar}(f',f). \tag{15}$$

In the limit of infinite population size, we find

$$\lim_{N\to\infty}\Phi_{\mathrm{dB},\bigstar}(f',f) = \frac{1-\omega}{1+\frac{f}{f'}}. \tag{16}$$

Therefore, deleterious mutations can fix in large populations with non-zero probability for any $\omega < 1$, see Fig. 2.

We now study the long-term evolution of the $\omega-\mathrm{dB}$ process on the star graph. In the limit of very large population size, the $\Psi_{\omega-\mathrm{dB},\bigstar}$ is given by

$$\Psi_{\omega-\mathrm{dB},\bigstar}(f',f) = \frac{\Phi_{\mathrm{dB},\bigstar}(f',f)}{\Phi_{\mathrm{dB},\bigstar}(f,f')} \approx \begin{cases} \dfrac{f'}{f}, & \text{if } 0\le\omega<1, \\[2ex] \left(\dfrac{f'}{f}\right)^2, & \text{if } \omega = 1. \end{cases} \tag{17}$$

Therefore, for any $\omega < 1$ the long-term steady state fitness statistics on the star graph is same as that of the parent moving case ($\omega = 0$). In another words, a very small proportion of parent moving is sufficient to significantly affect long-term evolution.

SUPPLEMENTARY NOTE 3: MATRIX APPROACH TO COMPUTE FIXATION PROBABILITY ON A RANDOM GRAPH

The matrix method solves the Markov chain for the fixation dynamics on a graph. This method is generally used to compute the fixation probability for an arbitrary connected graph [5]. The primary reference for this section is [6]. With states being the configurations of mutant and wild-type, the transition matrix $\mathbf{M}_{s\times s}$ is defined as

$$\mathbf{M}_{s\times s} = \begin{pmatrix} \mathbf{Q}_{t\times t} & \mathbf{R}_{t\times a} \\ \mathbf{0}_{a\times t} & \mathbf{I}_{a\times a} \end{pmatrix}, \tag{18}$$

where s denotes the total number of states and is equal to $t+a$, with t being the number of transient states and a being the number of absorbing states. $\mathbf{Q}$ is the transition probability matrix corresponding to the transitions among the transient states, while $\mathbf{R}$ represents transitions from the transient to the absorbing states. Since by definition there is no jump possible from an absorbing to the transient sector, the lower left matrix is a zero matrix. By similar reasoning, the lower right matrix is an identity matrix.

We denote the fundamental matrix as $\mathbf{F}$. It is equal to $\sum\limits_{n=0}^{\infty}\mathbf{Q}^n = (\mathbf{I}-\mathbf{Q})^{-1}$. The fixation probability to the absorbing state j when started in a transient state i is given by the relation,

$$\phi_{i,j} = (\mathbf{F}\cdot\mathbf{R})_{i,j}. \tag{19}$$

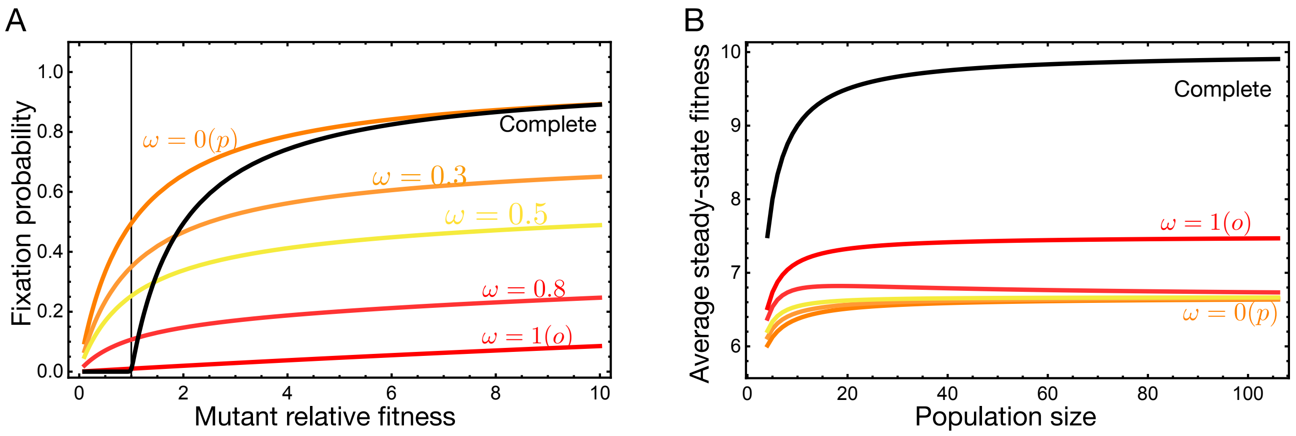

FIG. 2. **Fixation probabilities and long-term average fitness for the $\omega-$dB process on the star graph.** With probability ω offspring moves and with probability $1-\omega$ parent moves to vacant sites. In panel A, the fixation probability profiles for various ω values are shown for the dB process and panel B shows the average steady-state fitness for the corresponding ω values as a function of population size. The black curves corresponds to well-mixed population. We see that for the dB updating, the fixation probability for deleterious mutants in non-zero for intermediate ω values. Moreover, for large population sizes, the average long-term fitnesses for non-zero ω converge to the fitness of $\omega = 0$ process (parent moving) limit. Parameters for panel A: $N = 100$. Wild-type fitness is equal to one. Parameters for panel B: $f_{\rm min} = 0.1$ and $f_{\rm max} = 10$. The mutant fitness distribution is uniform.

Notice that the indices i, j etc., do not represent the number of mutants but the configurations themselves. The second index of the subscript can represent two absorbing states, every individual being the wild-type or the mutant type. It represents the position of the node where the initial mutant appears.

The fixation probability of a mutant to state j on a graph G with mutant initialisation $\mathcal{I}$ is equal to,

$$\Phi^{\mathcal{I}}_{j,G} = \sum_{i=0}^{N-1} p_i \phi_{i,j}. \tag{20}$$

The index i in the equation above corresponds to the states where the initial mutant appears at different positions on the graph.

1. *Transition matrix for the Bd process*

For a network of size N, we have the transition matrices of dimensions $2^N \times 2^N$. One can decrease the size of these matrices by considering the symmetries of the graph. This has been done in [7] where all the undirected connected networks of size four were considered. After considering the symmetries, the size of the transition matrices for the complete, diamond, and ring graph were reduced from 16 (2^4) to 5, 9, and 5, respectively. It is not straightforward to account for these symmetries for larger networks; thus, we work with maximum-size transition matrices. We compute the transition matrix for the Moran Bd dynamics with a

focus on undirected graphs and unweighted having symmetric adjacency matrices, $\mathbf{A}$. We work only with connected graphs as the fundamental matrix $\mathbf{F}$ becomes singular for the disconnected graphs.

The matrix element $\mathbf{M}_{ij}$ with $i \neq j$, the probability of going from state i to state j with $i \neq j$ is given as,

$$\mathbf{M}_{ij} = \begin{cases} \dfrac{1}{rn^i + N - n^i} \sum_k \tilde{a}_{km} \left[rn^i_k n^j_m + (1 - n^i_k)(1 - n^j_m) \right], & \text{if} \sum_s |n^i_s - n^j_s| = 1 \\ & \text{and}\, n^i_m \neq n^j_m, \\ 0, & \text{otherwise.} \end{cases} \tag{21}$$

where every node of the configuration i can either take a value 0 (for wild-type) or 1 (for mutant). The number of mutants in state i is given by $n^i = \sum_{k=0}^{N-1} n^i_k$. Here, we have also introduced $\tilde{a}_{km} = \dfrac{a_{km}}{\sum_l a_{kl}}$. The diagonal elements $\mathbf{M}_{ii}$ are equal to $1 - \sum_{j \neq i} \mathbf{M}_{ij}$.

2. Transition matrix for dB process

Like the Bd process, here we write down the transition matrix for the dB process. The matrix element $\mathbf{M}_{ij}$ with $i \neq j$ is given as :

$$\mathbf{M}_{ij} = \begin{cases} \dfrac{1}{N} \sum_k \dfrac{a_{km} \left[rn^i_k n^j_m + (1 - n^i_k)(1 - n^j_m) \right]}{r \sum_l a_{lm} n^i_l + \sum_l a_{lm}(1 - n^i_l)}, & \text{if} \sum_s |n^i_s - n^j_s| = 1 \,\text{and}\, n^i_m \neq n^j_m, \\ 0, & \text{otherwise.} \end{cases} \tag{22}$$

SUPPLEMENTARY NOTE 4: LONG-TERM EVOLUTION ON REGULAR GRAPHS

Under the Moran dB updating, regular graphs have different fixation probability profiles compared to the complete graph [8]. It is in contrast to the Moran Birth-death (Bd) rule, where according to the isothermal theorem [9, 10] all the regular graphs have the same fixation probability profiles as the well-mixed population. For example, under Moran dB updating, the cycle graph has the fixation probability [11],

$$\Phi_\circ(f', f) = \frac{2\left(1 - \frac{f}{f'}\right)}{3 - \frac{f}{f'} + \left(1 - 3\frac{f}{f'}\right)\left(\frac{f}{f'}\right)^{N-2}}. \tag{23}$$

Under the Moran dB updating, the cycle graph is a suppressor of fixation [11], because $\Phi_\circ(f', f) < \Phi_C(f', f)$ for all pairs of f' and f (ignoring the neutral point $f' = f$, where

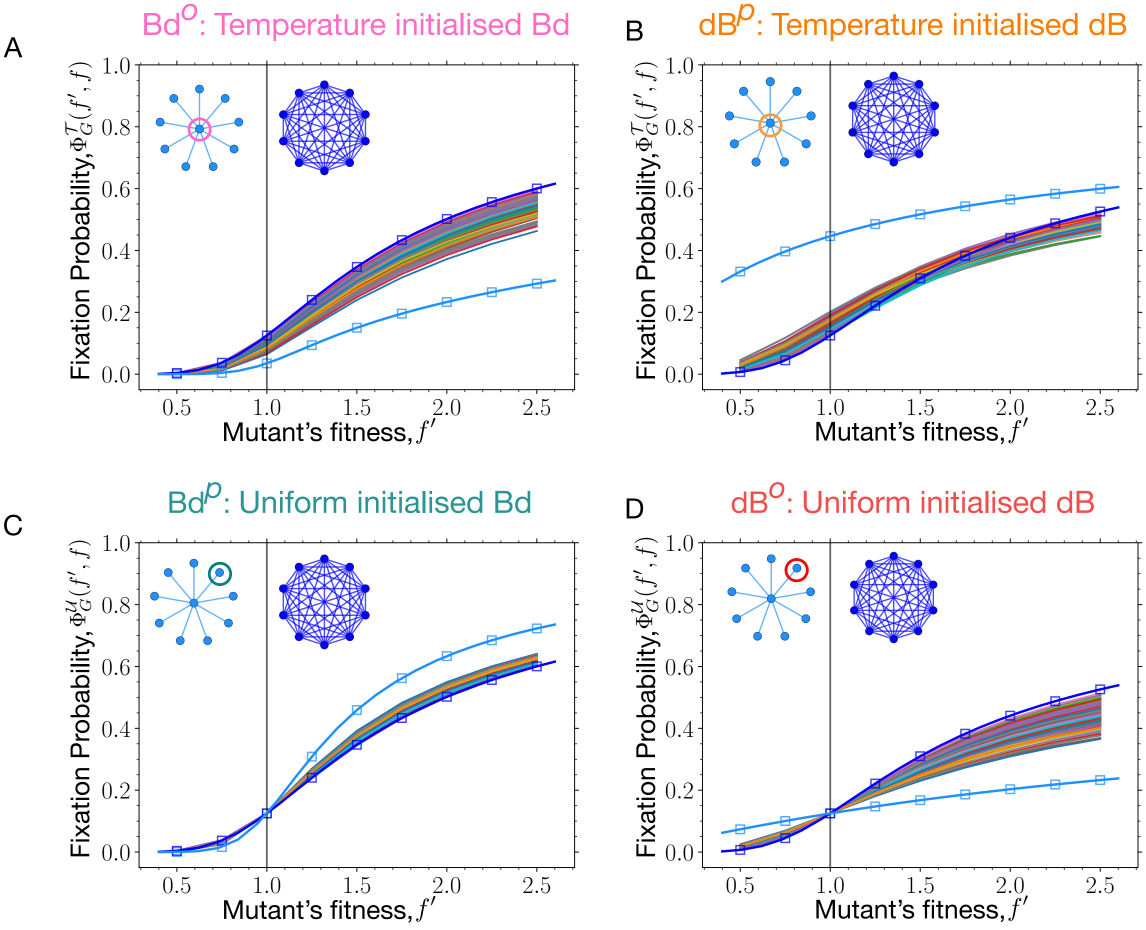

FIG. 3. **Fixation probability profiles of random graphs.** The relative fitness values of the mutant are chosen to be, 0.5, 0.75, 1, 1.25, 1.5, 1.75, 2, 2.25 and 2.5 with wild-type fitness being 1. The fixation probability profiles for the complete graph (in dark blue) and the star graph (in bright blue) in both numerical (square markers) and analytical (solid lines) form are shown. The other colored curves correspond to the fixation probability profiles for connected Erdős Rényi random graphs of size 8 generated with a probability of link connection $p = 0.5$ (sampling 500 times). A) Most of the random graphs are suppressors of fixation under temperature initialised Bd updating. B) Most graphs are piecewise amplifiers of fixation under dB^p updating i.e. the graphs have higher fixation probability for a mutant with fitness below a certain value, f^*, and lower fixation probability beyond f^*. We observe that beyond $f' \approx 1.5$, the fixation probabilities become lower than the fixation probability on the well-mixed population. C) Most of the graphs are amplifiers of selection under (Bd^p) uniform initialised Bd updating, and D) Most of the graphs are suppressors of selection under (dB^o) uniform initialised dB updating.

$\Phi_\circ(f', f) = \Phi_C(f', f) = \frac{1}{N}$), see Fig. 4 B. The ratio of fixation probabilities entering the

steady-state detailed balance solution, however, is the same as complete graph,

$$
\begin{aligned}
\Psi_\circ(f', f) &= \frac{\Phi_\circ(f', f)}{\Phi_\circ(f, f')}, \\
&= \frac{2\left(1-\frac{f}{f'}\right)}{3-\frac{f}{f'}+\left(1-3\frac{f}{f'}\right)\left(\frac{f}{f'}\right)^{N-2}} \cdot \frac{3-\frac{f'}{f}+\left(1-3\frac{f'}{f}\right)\left(\frac{f'}{f}\right)^{N-2}}{2\left(1-\frac{f'}{f}\right)}, \\
&= \frac{f'-3f+(3f'-f)\left(\frac{f'}{f}\right)^{N-2}}{3f'-f+(f'-3f)\left(\frac{f}{f'}\right)^{N-2}}, \\
&= \left(\frac{f'}{f}\right)^{N-2}.
\end{aligned} \tag{24}
$$

Because $\Psi_\circ(f', f)$ is a power law, the Moran dB origin-fixation dynamics on the cycle graph is reversible. Moreover,

$$
P^*_\circ(f) = P^*_C(f). \tag{25}
$$

Consequently, $\langle f \rangle^*_\circ = \langle f \rangle^*_C$.

What did we just learn? We learnt that the cycle graph despite being a suppressor of fixation, attains the same average fitness in the mutation-selection balance as the complete graph, see Fig. 4 D. In the limit of $N \to \infty$,

$$
\lim_{N\to\infty} \Phi_\circ(f', f) = \begin{cases} \frac{2\left(\frac{f'}{f}-1\right)}{3\frac{f'}{f}-1}, & \text{if } f' > f, \\ 0 & \text{otherwise.} \end{cases} \tag{26}
$$

Now, $\frac{2\left(\frac{f'}{f}-1\right)}{3\frac{f'}{f}-1} < 1 - \frac{f}{f'}$ for all $f' > f$, therefore, in the limit of very large population sizes, the cycle graph is less likely to fix beneficial mutants than the complete graph, see Fig. 4 A. Yet the cycle and the complete graph attain the same steady-state average fitness for all population sizes. It happens because the cycle graph is better at rejecting deleterious mutants than the complete graph. The ability of the cycle graph to prevent the fixation of disadvantageous mutants compensates for its lower probability to fix beneficial mutants in a way that $\Psi_\circ(f', f)$ becomes equal to $\Psi_C(f', f)$.

We also explore the steady-state statistics of the long-term dB mutation-selection dynamics on the two dimensional lattice with periodic boundary conditions. With each node having k neighbors, the fixation probability of a mutant to fix on the 2d lattice is [8],

$$
\Phi_{2d}(f', f) \approx \frac{k\left(1-\frac{f}{f'}\right)}{k\left(1-\frac{f}{f'}\right)+\left(1-\left(\frac{f}{f'}\right)^{N-2}\right)\left(1+(k-1)\frac{f}{f'}\right)}. \tag{27}
$$

We focus on the case of $k = 4$. The ratio of fixation probabilities takes the form,

$$
\Psi_{2d}(f', f) = \frac{\Phi_{2d}(f', f)}{\Phi_{2d}(f, f')} \approx \left(\frac{f'}{f}\right)^{N-2} \frac{5-\frac{f'}{f}-\left(\frac{f'}{f}\right)^{N-2}\left(3\frac{f'}{f}+1\right)}{3+\frac{f'}{f}+\left(\frac{f'}{f}\right)^{N-2}\left(1-5\frac{f'}{f}\right)}. \tag{28}
$$

From Fig. 4 A and C, it is clear that the fixation probability profile of the 2d lattice is different from the well-mixed population, but the graph category to which the 2d lattice belongs is not clear. This could be due to the approximation made in Ref. [8] to compute Eq. 27. As a result, for small N, the 2d lattice attains different (lower) steady-state average fitness in the mutation-selection balance than the complete and cycle graph. However, with an increase in population size, the steady-state average fitness attained by the 2d lattice asymptotes to the one attained by the well-mixed population, see Fig. 4 D. This can be understood by performing the large N expansion on $\Psi_{2d}(f', f)$ yielding,

$$\Psi_{2d}(f', f) \approx \begin{cases} \left(\frac{f'}{f}\right)^{N-2} \frac{3\frac{f'}{f}+1}{5\frac{f'}{f}-1}, & \text{if } f' > f, \\ \left(\frac{f'}{f}\right)^{N-2} \frac{5-\frac{f'}{f}}{3+\frac{f'}{f}}, & \text{otherwise.} \end{cases} \tag{29}$$

For large population sizes, $\Psi_{2d} \sim \left(\frac{f'}{f}\right)^{N-2}$, $P^*_{2d}(f)$ and therefore, $\langle f \rangle^*_{2d}$ take the same limit as the complete graph. In the argument made, we have assumed reversibility to hold. In the subsequent section, we justify the assumption.

From the above two case studies, we have learnt that although the complete graph, the cycle graph, and the 2d lattice behave differently at fixation time scales under dB updating [8], their steady-state statistics for the long-term Moran dB origin-fixation dynamics are identical.

SUPPLEMENTARY NOTE 5: REVERSIBILITY

The primary reference for this section is [12]. Using the Kolmogorov criterion [13], the neutral Moran origin-fixation dynamics turns out to be reversible. With this, the origin-fixation dynamics under selection is reversible if $\Psi^{\mathcal{I}}_G(r)$ is a power law,

$$\Psi^{\mathcal{I}}_G(r) = r^{\nu}, \tag{30}$$

where r is used as a shorthand for $\frac{f'}{f}$, and ν is given by the relation,

$$\nu = 2\left.\frac{\frac{\mathrm{d}\Phi^{\mathcal{I}}_G}{\mathrm{d}r}}{\Phi^{\mathcal{I}}_G(r)}\right|_{r=1}. \tag{31}$$

In general, $\Psi^{\mathcal{I}}_G(r)$ need not be a power law. Therefore, to check the scope for reversibility a logarithmic expansion is performed on $\Psi^{\mathcal{I}}_G(r)$,

$$\begin{aligned} \log \Psi^{\mathcal{I}}_G(r) &= \sum_{j=0}^{\infty} \frac{c_{2j+1}}{(2j+1)!} (\log r)^{2j+1}, \\ &= c_1 \log r \left[1 + \frac{1}{c_1} \sum_{j=1}^{\infty} \frac{c_{2j+1}}{(2j+1)!} (\log r)^{2j}\right], \end{aligned} \tag{32}$$

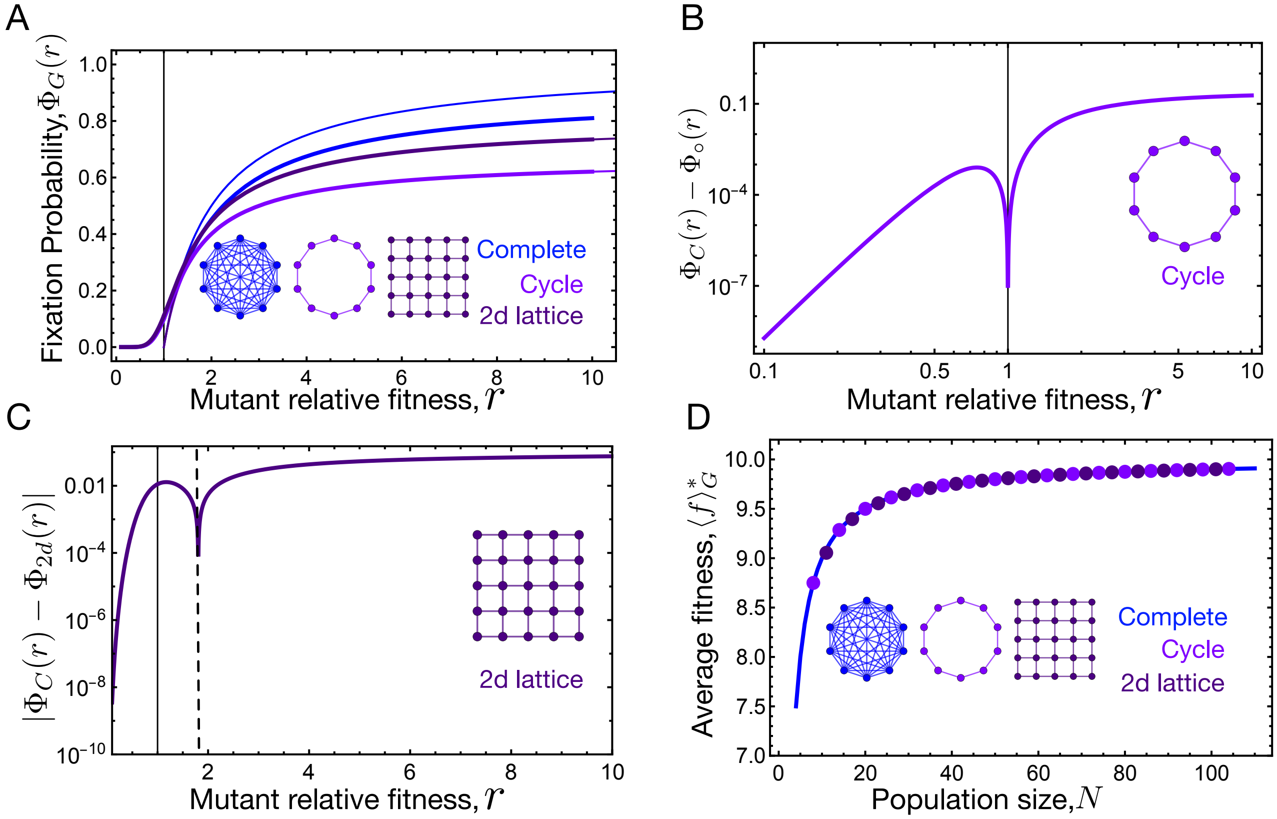

FIG. 4. **Regular graph: dB fixation probability and long-term evolution.** A) Fixation probability for regular graphs – the cycle graph, the 2d lattice with periodic boundaries and the complete graph – under dB updating are shown. Thick lines correspond to population size $N = 10$. Thin lines represents $\lim_{N\to\infty} \Phi_G(f', f)$. At fixation time scales, regular graphs behave differently. B) The cycle graph is a suppressor of fixation under dB updating. C) For small N (N=10 here), the 2d lattice behaves like a suppressor of selection. $\Phi_C - \Phi_{2d}$ is negative for mutant fitnesses to the left of the dashed line, and positive otherwise. D) The cycle graph being a suppressor of fixation, attains the same steady-state average fitness in the mutation selection balance as the complete graph. For large N, $2d$ lattice attains the same fitness as the other graphs. At longer time scales, regular graphs have identical steady-state statistics contrary to their differences at shorter fixation time scales. Parameters: mutant fitness distribution, $\rho(f', f) = \frac{1}{f_{\max} - f_{\min}}$ with $f_{\min} = 0.1$ and $f_{\max} = 10$.

where, $c_i = \left.\frac{\mathrm{d}^i \log \Psi_G^{\mathcal{I}}}{\mathrm{d}(\log r)^i}\right|_{r=1}$ and $c_1 = \nu$. This can be seen as following,

$$
\begin{aligned}
c_1 &= \left.\frac{\mathrm{d}\log \Psi_G^{\mathcal{I}}}{\mathrm{d}\log r}\right|_{r=1}, \\
&= \left.\frac{\mathrm{d}}{\mathrm{d}\log r}\log\frac{\Phi_G^{\mathcal{I}}(r)}{\Phi_G^{\mathcal{I}}(\frac{1}{r})}\right|_{r=1}, \\
&= \left.\left(\frac{\mathrm{d}\log r}{\mathrm{d}r}\right)^{-1}\frac{\mathrm{d}}{\mathrm{d}r}\log\frac{\Phi_G^{\mathcal{I}}(r)}{\Phi_G^{\mathcal{I}}(\frac{1}{r})}\right|_{r=1}, \\
&= \left.\frac{r}{\frac{\Phi_G^{\mathcal{I}}(r)}{\Phi_G^{\mathcal{I}}(\frac{1}{r})}}\frac{\mathrm{d}}{\mathrm{d}r}\left(\frac{\Phi_G^{\mathcal{I}}(r)}{\Phi_G^{\mathcal{I}}(\frac{1}{r})}\right)\right|_{r=1}, \\
&= \left.\left(\frac{r}{\Phi_G^{\mathcal{I}}(r)}\frac{\mathrm{d}\Phi_G^{\mathcal{I}}}{\mathrm{d}r} + \frac{1}{r\Phi_G^{\mathcal{I}}(\frac{1}{r})}\frac{\mathrm{d}\Phi_G^{\mathcal{I}}}{\mathrm{d}r}\right)\right|_{r=1}, \\
&= 2\frac{\left.\frac{\mathrm{d}\Phi_G^{\mathcal{I}}}{\mathrm{d}r}\right|_{r=1}}{\Phi_G^{\mathcal{I}}(1)}, \\
&= \nu.
\end{aligned} \tag{33}
$$

The series 32 contains only odd terms because, by definition, $\Psi_G^{\mathcal{I}}(1/r) = \Psi_G^{\mathcal{I}}(r)^{-1}$. The second term of the series gives us the conservative estimate of the range of fitness values for which the origin-fixation dynamics is reversible, $(r_0^{-1},\, r_0)$ and r_0 is found by setting a tolerance ε,

$$
\frac{|c_3|}{6\nu}(\log r_0)^2 = \varepsilon. \tag{34}
$$

For the complete and the cycle graph, Ψ_G is a strict power-law with $c_3 = 0$, which is why $r_0 \to \infty$ for these structures. However, for the 2d lattice, $\frac{|c_3|}{6\nu}$ saturates to a finite value at larger N, see Fig. 5, as both c_3 and ν scales as N at large population sizes.

$$
\begin{aligned}
c_3 &= \frac{(N-2)^2(4N^2-9N+6)}{16(N-1)^3} \approx \frac{N}{4}, \\
\nu &= N - \frac{5}{2} + \frac{1}{2(N-1)}.
\end{aligned} \tag{35}
$$

This makes r_0 finite with a value approximately equal to 3 for $\epsilon = 0.05$ and the range of fitness values where the Moran dB origin-fixation dynamics is reversible is $r \in (0.33, 3)$. Above, we have used the relation,

$$
\begin{aligned}
c_3 &= \left.\frac{\mathrm{d}^3\log \Psi_G^{\mathcal{I}}}{\mathrm{d}(\log r)^3}\right|_{r=1}, \\
&= \left.r\frac{\mathrm{d}}{\mathrm{d}r}\left(r\frac{\mathrm{d}}{\mathrm{d}r}\left(r\frac{\mathrm{d}\log\Psi_G^{\mathcal{I}}}{\mathrm{d}r}\right)\right)\right|_{r=1}.
\end{aligned} \tag{36}
$$

Similarly, $\frac{|c_3|}{6\nu} \sim \frac{1}{N}$ for the temperature initialised star graph under dB (dB^p) dynamics. As a result $r_0 \to \infty$ in the limit $N \to \infty$. The same result holds for the star graph under dB^o dynamics.

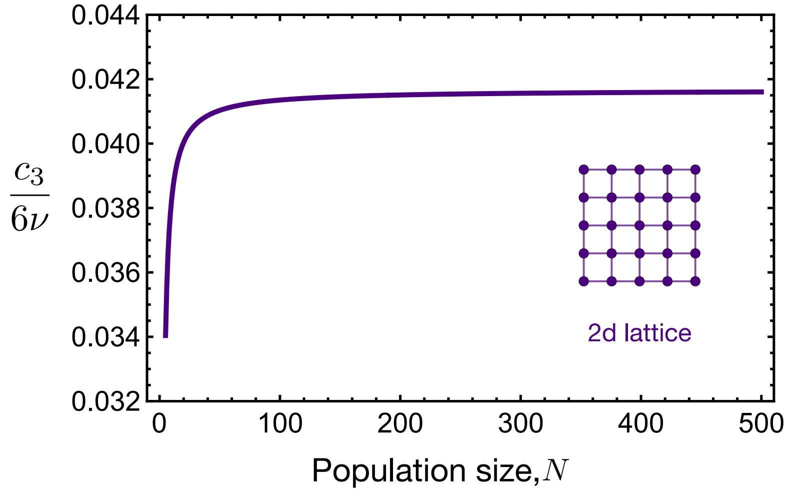

FIG. 5. **Reversible Moran dB origin-fixation dynamics on the 2d lattice.** Coefficient $\frac{|c_3|}{6\nu}$ of the second term of Eq. 32 saturates to finite value for large N, which results in the range of fitness values where the origin-fixation dynamics is reversible to be $r \in (0.33, 3)$.

SUPPLEMENTARY NOTE 6: STANDARD DEVIATION IN THE STEADY-STATE FITNESS DISTRIBUTION

In this section, we derive the expressions for the standard deviation in fitness in the steady-state. For the complete graph we know that,

$$P_C(f) = \frac{N-1}{f_{\max}^{N-1} - f_{\min}^{N-1}} f^{N-2} \quad \text{and} \quad \langle f \rangle_C = \frac{N-1}{N} \frac{f_{\max}^{N} - f_{\min}^{N}}{f_{\max}^{N-1} - f_{\min}^{N-1}}, \tag{37}$$

and

$$\langle f^2 \rangle_C = \frac{N-1}{N+1} \frac{f_{\max}^{N+1} - f_{\min}^{N+1}}{f_{\max}^{N-1} - f_{\min}^{N-1}}. \tag{38}$$

The variance turns out to be

$$\begin{aligned} \mathrm{Var}_C &= \langle f^2 \rangle_C - (\langle f \rangle_C)^2, \\ &= f_{\min}^2 \left[\frac{N-1}{N+1} \frac{\left(\frac{f_{\max}}{f_{\min}}\right)^{N+1} - 1}{\left(\frac{f_{\max}}{f_{\min}}\right)^{N-1} - 1} - \left(\frac{N-1}{N}\right)^2 \left(\frac{\left(\frac{f_{\max}}{f_{\min}}\right)^{N} - 1}{\left(\frac{f_{\max}}{f_{\min}}\right)^{N-1} - 1} \right)^2 \right], \\ &\approx f_{\max}^2 \left(\frac{N-1}{N+1} - \left(\frac{N-1}{N}\right)^2 \right), \\ &\approx \frac{f_{\max}^2}{N^2}. \end{aligned} \tag{39}$$

The above approximation holds for large population sizes. Therefore, the standard deviation in fitness for the complete scales as $\sim 1/N$. For the star graph under dB^o dynamics, in the limit of large N

$$P_{\mathrm{dB}^o,\bigstar}(f) = \frac{3f^2}{f_{\max}^3 - f_{\min}^3} \quad \text{and} \quad \langle f \rangle_{\mathrm{dB}^o,\bigstar} = \frac{3}{4}\frac{f_{\max}^4 - f_{\min}^4}{f_{\max}^3 - f_{\min}^3}, \tag{40}$$

and

$$\langle f^2 \rangle_{\mathrm{dB}^o,\bigstar} = \frac{3}{5}\frac{f_{\max}^5 - f_{\min}^5}{f_{\max}^3 - f_{\min}^3}. \tag{41}$$

For the variance, we have

$$\begin{aligned} \mathrm{Var}_{\mathrm{dB}^o,\bigstar} &= \langle f^2 \rangle_{\mathrm{dB}^o,\bigstar} - \left(\langle f \rangle_{\mathrm{dB}^o,\bigstar}\right)^2, \\ &\approx \frac{3}{80} f_{\max}^2. \end{aligned} \tag{42}$$

The standard deviation for the dB^o star graph is independent of N and asymptotes to a finite value. Similarly, for the dB^p star graph, for $N \gg 1$ we have

$$\begin{aligned} \mathrm{Var}_{\mathrm{dB}^p,\bigstar} &= \langle f^2 \rangle_{\mathrm{dB}^p,\bigstar} - \left(\langle f \rangle_{\mathrm{dB}^p,\bigstar}\right)^2, \\ &\approx \frac{1}{18} f_{\max}^2, \end{aligned} \tag{43}$$

which is again independent of N. Note that for large N, $\mathrm{Var}_{\mathrm{dB}^p,\bigstar} > \mathrm{Var}_{\mathrm{dB}^o,\bigstar} > \mathrm{Var}_C$. The order of fluctuations can be understood from the large N limit effective population size of the three structures– N for the complete graph, 2 for the dB^o star graph and 1 for the dB^p star graph.

Under Bd updating, we have seen that $\Psi_\bigstar$ is same for both the Bd^o (Bd temperature initialisation) and Bd^p (Bd uniform initialisation) star graph. Consequently, the Bd^o and Bd^p have the same steady-state statistics and thus, variance in the steady-state,

$$\begin{aligned} \mathrm{Var}_{\mathrm{Bd}^o,\bigstar} &= \mathrm{Var}_{\mathrm{Bd}^p,\bigstar}, \\ &= \langle f^2 \rangle_{\mathrm{Bd}^p,\bigstar} - \left(\langle f \rangle_{\mathrm{Bd}^p,\bigstar}\right)^2, \\ &= \left[\frac{2N-1}{2N+1}\frac{f_{\max}^{2N+1} - f_{\min}^{2N+1}}{f_{\max}^{2N-1} - f_{\min}^{2N-1}} - \left(\frac{2N-1}{2N}\frac{f_{\max}^{2N} - f_{\min}^{2N}}{f_{\max}^{2N-1} - f_{\min}^{2N-1}}\right)^2\right], \\ &\approx f_{\max}^2 \left[\frac{2N-1}{2N+1} - \left(\frac{2N-1}{2N}\right)^2\right], \\ &\approx \frac{f_{\max}^2}{4N^2}. \end{aligned} \tag{44}$$

The above approximation holds for large N. Therefore, the standard deviation for the star graph under Moran origin-fixation Bd updating scales as $\sim \dfrac{1}{2N}$.

SUPPLEMENTARY NOTE 7: LONG-TERM EVOLUTION ON DISCRETE FITNESS SPACE

To study Bd and dB long-term dynamics on random graphs we define a Markov chain. To proceed we first discretise the fitness space. The states are labelled with integer values from $0, 1, \cdots, z$. For $1 \leq i \leq z-1$, the fitness of state i is equal to $f_i = f_{\min} + i \cdot \kappa$. The boundaries fitness are $f_0 = f_{\min}$ and $f_z = f_{\max}$. The long-term dynamics on a graph G is a Markov chain obeying the equation,

$$\mathbf{P}_G(t+1) = \mathbf{P}_G(t) \cdot \mathbf{T}_G, \tag{45}$$

where $\mathbf{P}_G(t) = (P_{G,0}(t), P_{G,1}(t), \cdots, P_{G,z}(t))$ with $P_{G,i}(t)$ being the probability for the population to be in fitness state f_i at time step t. The transition matrix $\mathbf{T}_G$ on the fitness space is given as,

$$T_{G,ij} = \begin{cases} \frac{1}{2}\Phi_G^{\mathcal{I}}(f_{j+1}, f_j), & \text{if}\, i = j+1 \,\text{and}\, 0 \leq j < z, \\ \frac{1}{2}\Phi_G^{\mathcal{I}}(f_{j-1}, f_j), & \text{if}\, i = j-1 \,\text{and}\, 0 < j \leq z, \\ 1 - \sum\limits_{k \neq i} T_{G,ki}, & \text{if}\, i = j. \end{cases} \tag{46}$$

Matrix $\mathbf{T}_G$ is a positive and an irreducible matrix, therefore we can find the steady-state distribution $\mathbf{P}^*_G$ using the Perron-Frobenius theorem [14]. $\mathbf{P}^*_G$ is the left eigenvector of the matrix $\mathbf{T}_G$ corresponding to eigenvalue 1,

$$\mathbf{P}^*_G \cdot \mathbf{T}_G = 1 \cdot \mathbf{P}^*_G. \tag{47}$$

The steady-state average fitness is then,

$$\langle f \rangle^*_G = \boldsymbol{f} \cdot \mathbf{P}^*_G, \tag{48}$$

where $\boldsymbol{f} = (f_{\min}, f_1, f_2, \cdots, f_{\max})$ is the fitness vector.

SUPPLEMENTARY NOTE 8: CRITERION FOR A GRAPH TO HAVE HIGHER STEADY-STATE FITNESS THAN THE COMPLETE GRAPH

Just by looking at the $\Phi_G^{\mathcal{I}}(f', f)$, it is not easy to predict if the graph G will attain higher long-term average fitness than the complete graph in the mutation-selection balance of the Moran origin-fixation dynamics. The useful quantity for that purpose is the ratio of fixation probabilities, $\Psi_G^{\mathcal{I}}(f', f)$ as defined in Eq. 12 (main text), which allows us to obtain a sufficient condition. Let us consider two graphs, G_1 and G_2, and denote the respective steady-state probability density functions for the Moran origin-fixation dynamics on these structures by $P^*_{G_1}(f)$ and $P^*_{G_2}(f)$.

Proposition: If the continuous density functions $P^*_{G_1}(f)$ and $P^*_{G_2}(f)$ intersect exactly once, then

$$\Psi_{G_1}^{\mathcal{I}}(f', f_{\max}) < \Psi_{G_2}^{\mathcal{I}}(f', f_{\max}), \text{ for all } f', \tag{49}$$

is a sufficient condition for $\langle f \rangle^*_{G_1} > \langle f \rangle^*_{G_2}$.

Proof: From the condition (49) and Eq. 12 of the main text, it follows that

$$P^*_{G_1}(f_{\max}) > P^*_{G_2}(f_{\max}). \tag{50}$$

Using this fact, and denoting $\hat{f}$ as the unique point at which the the functions intersect,

$$P^*_{G_1}(f) = \begin{cases} P^*_{G_2}(f) + \varepsilon^<(f) & \text{if } f \le \hat{f}, \quad \text{with } \varepsilon^<(f) < 0 \\ P^*_{G_2}(f) + \varepsilon^>(f) & \text{otherwise,} \quad \text{with } \varepsilon^>(f) > 0. \end{cases}$$

Then, from the normalization of the probability density functions, it follows that

$$-\int\limits_{f_{\min}}^{\hat{f}} \mathrm{d}f\, \varepsilon^< = \int\limits_{\hat{f}}^{f_{\max}} \mathrm{d}f\, \varepsilon^>. \tag{51}$$

Now, note that

$$\langle f\rangle^*_{G_1} = \langle f\rangle^*_{G_2} + \int\limits_{f_{\min}}^{\hat{f}} \mathrm{d}f\, f\varepsilon^< + \int\limits_{\hat{f}}^{f_{\max}} \mathrm{d}f\, f\varepsilon^> \tag{52}$$

$$> \langle f\rangle^*_{G_2} + \hat{f}\int\limits_{f_{\min}}^{\hat{f}} \mathrm{d}f\, \varepsilon^< + \hat{f}\int\limits_{\hat{f}}^{f_{\max}} \mathrm{d}f\, \varepsilon^> \tag{53}$$

$$= \langle f\rangle^*_{G_2}, \tag{54}$$

where the inequality follows straightforwardly from the signs of the integrands and the limits of the integrals, and the last equality follows from (51). This completes the proof. □
The criterion $\Psi^{\mathcal{I}}_G(f', f_{\max}) < \Psi_C(f', f_{\max})$ for all f', is naturally satisfied by amplifiers of selection: It follows directly from the definition of amplifiers of selection, which are better at preventing the fixation of deleterious mutations and fixing advantageous mutants. Therefore, for an amplifier of selection,

$$\underbrace{\frac{\Phi^{\mathcal{I}}_{AoS}(f', f_{\max})}{\Phi^{\mathcal{I}}_{AoS}(f_{\max}, f')}}_{\Psi^{\mathcal{I}}_{AoS}(f', f_{\max})} < \underbrace{\frac{\Phi_C(f', f_{\max})}{\Phi_C(f_{\max}, f')}}_{\Psi_C(f', f_{\max})} \text{ for all } f'. \tag{55}$$

Subscript AoS denotes an amplifier of selection. Similarly, for a suppressor of selection,

$$\underbrace{\frac{\Phi^{\mathcal{I}}_{SoS}(f', f_{\max})}{\Phi^{\mathcal{I}}_{SoS}(f_{\max}, f')}}_{\Psi^{\mathcal{I}}_{SoS}(f', f_{\max})} > \underbrace{\frac{\Phi_C(f', f_{\max})}{\Phi_C(f_{\max}, f')}}_{\Psi_C(f', f_{\max})} \text{ for all } f'. \tag{56}$$

Subscript SoS denotes a suppressor of selection.

For suppressors of fixation, it is not obvious if they satisfy the criterion to attain higher average steady-state fitness in the mutation-selection balance. Suppressors of fixation have a lower probability of fixing beneficial mutations. As a result, $[\Phi^{\mathcal{I}}_{SoF}(f_{\max}, f')]^{-1} >$

$[\Phi_C(f_{\max}, f')]^{-1}$ (here SoF denotes a suppressor of fixation). However, they are also good in preventing the fixation of deleterious mutants, $\Phi^{\mathcal{I}}_{SoF}(f', f_{\max}) < \Phi_C(f', f_{\max})$. This means that a suppressor of fixation can satisfy the criterion 49 by compensating for its lower probability of fixation of beneficial mutants by rejecting deleterious mutants more efficiently. An example is presented in ref. [15]. Note that the criterion 49 holds for any update rule.

Let us apply the derived sufficient condition to the complete graph, dB^o and dB^p star graph. From Fig. 6, we see that the steady-state probability density functions for any two graphs intersect at only one point, and thus we can use our criteria here. From Eqs. 14, 19, and 23, we have

$$\Psi^{\mathcal{I}}_C(f', f_{\max}) < \Psi_{\mathrm{dB}^o,\bigstar}(f', f_{\max}) < \Psi_{\mathrm{dB}^p,\bigstar}(f', f_{\max}), \tag{57}$$

therefore, $\langle f \rangle^*_C > \langle f \rangle^*_{\mathrm{dB}^o,\bigstar} > \langle f \rangle^*_{\mathrm{dB}^p,\bigstar}$.

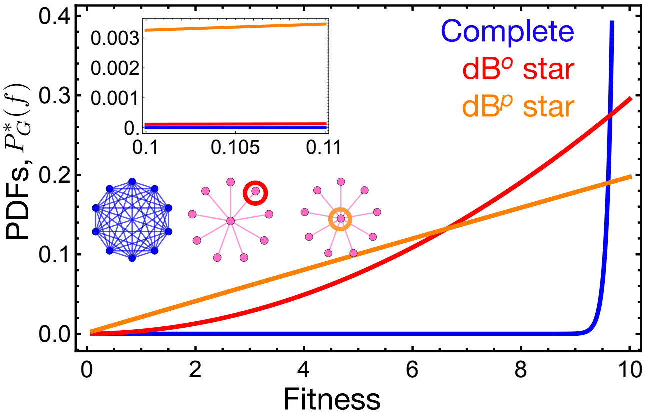

FIG. 6. **A pair of probability density functions intersect at one fitness point.** The steady-state probability density functions (PDFs) for the complete graph, the dB^o and the dB^p star graph are shown. Firstly, the PDFs are monotonically increasing w.r.t fitness. Secondly, any pair of PDFs intersects at one point. Thus the sufficient condition 49 gives the ordering of average fitnesses. Parameters: $N = 100$, $f_{\min} = 0.1$, $f_{\max} = 10$, $\rho(f', f) = \frac{1}{f_{\max} - f_{\min}}$.

SUPPLEMENTARY NOTE 9: EFFECTS OF DELETERIOUS MUTANTS ON THE INITIAL PHASE OF THE LONG-TERM DYNAMICS

So far we have studied the effect of preventing/fixing deleterious mutants on the steady-state fitness statistics of graph. In this section, we explore the role of deleterious mutant regime on the initial phase of the mutation-selection dynamics, particularly the average selection coefficient of the first substitution given that the initial fitness is f,

$$\frac{\Delta_G f}{f} = \int \mathrm{d}f' \, \frac{f' - f}{f} \Phi^{\mathcal{I}}_G(f', f)\rho(f', f). \tag{58}$$

This quantity is also equal to the instantaneous rate of evolution [16], [17]. From Fig. 7 A, we see that the mean selection coefficient decreases as the population is started with initial population fitness closer to the average steady-state fitness. The mean selection coefficient

is anticipated to be positive if the initial fitness is below the average steady-state fitness and negative if started with higher fitness values. However, in ref. [18], for the complete graph it has been shown that the mean selection coefficient of the first substitution is negative even if the population is initialised with fitness below the average steady-state fitness. We observe this effect to be more pronounced for the case of dB^p star graph. We investigate further by working out the large N case.

In the limit of $N \to \infty$, for the dB^p star graph under Moran dB^o updating and uniform mutational fitness distribution, we have

$$\begin{aligned} \frac{\Delta_{\mathrm{dB}^p,\bigstar} f}{f} &= \frac{1}{f_{\max} - f_{\min}} \int \mathrm{d}f' \, \frac{f' - f}{f} \frac{f'}{f + f'} \\ &= \frac{f_{\max} + f_{\min} - 4f}{2f} - \frac{1}{f_{\max} - f_{\min}} 2f \log\left(\frac{f_{\max} + f}{f_{\min} + f}\right). \end{aligned} \tag{59}$$

The value of f for which the mean selection coefficient (right hand side of the above equation) goes to zero is obtained numerically. The difference of the obtained fitness value and the average steady-state fitness for the dB^p graph, δf is shown in Fig. 7 B. For the parameters we use throughout the manuscript, the δf for the dB^p star graph is finite, even in the limit of $N \to \infty$. This means that if the long-term mutation-selection dynamics is initiated with fitness value in between $\langle f \rangle^*_{\mathrm{dB}^p,\bigstar} - \delta f$ and $\langle f \rangle^*_{\mathrm{dB}^p,\bigstar}$, the first mutation to fix on average is deleterious. Therefore, the corresponding average fitness trajectories does not have monotonically increasing fitnesses. For the complete graph, in the limit of $N \to \infty$,

$$\begin{aligned} \frac{\Delta_C f}{f} &= \frac{1}{f_{\max} - f_{\min}} \int \mathrm{d}f' \, \frac{f' - f}{f} \frac{f' - f}{f'} \\ &= \frac{1}{f_{\max} - f_{\min}} \left(-2f_{\max} + \frac{f_{\max}^2}{2f} + \frac{3f}{2} + f \log \frac{f_{\max}}{f} \right) \end{aligned} \tag{60}$$

The r.h.s in the above equation goes to 0 for $f = f_{\max}$. Therefore, while δf is non-zero for finite population sizes, in the limit of $N \to \infty$, $\delta f = 0$ for the complete graph.

SUPPLEMENTARY NOTE 10: AMPLIFIER OF FIXATION AND SUPPRESSOR OF FIXATION IN A METAPOPULATION MODEL

The amplifiers and suppressors of fixation are not solely restricted to one node one individual models, but can also be found in the network structured metapopulations as observed in the supplementary information of Marrec et al., Ref. [19]. For completeness, we summarize their argument here. The model in ref. [19] assumes time scale separation. The model has been analysed in the low migration rate regime where each node/deme is mainly in a monomorphic state. That is, when an individual migrates to a neighboring patch with a different type, either the migrating individual fixes or goes extinct before the next migration event occurs. The wild-type and the mutant type are assumed to have non-zero death rates, as a consequence of which the population sizes of fully mutant and wild-type demes are different. Here we assume that for both the individual types, the death rate is incorporated with the birth rate to give an effective growth rate. In this way, the population size for the mutant and wild-type deme is the same. The number of leaf demes is denoted by d. The probability that an individual from a given leaf deme migrates to the center deme is

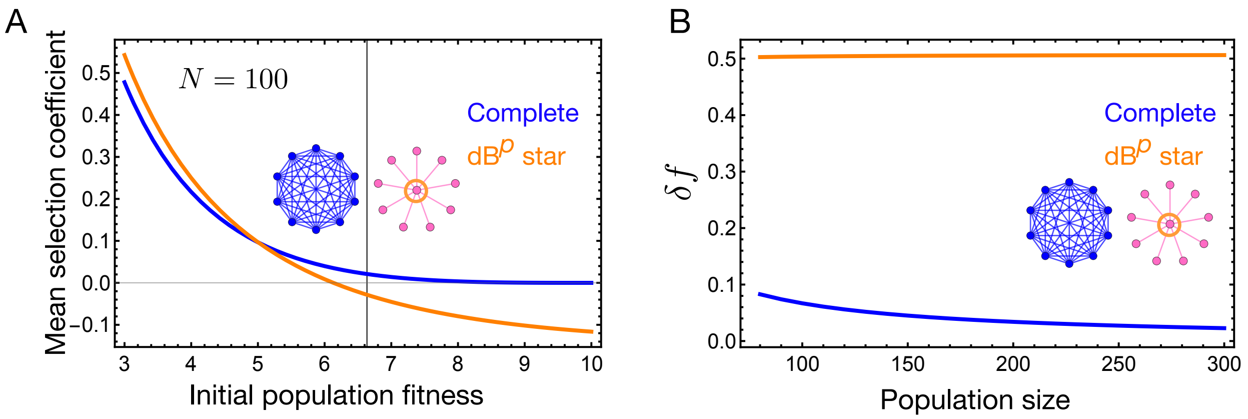

FIG. 7. **Mean selection coefficient of the first substitution.** The black grid line in A) corresponds to the average steady-state fitness of the dB^p star graph. The mean selection coefficient is negative even if the population's initial fitness is below the steady-state average fitness, thus leading to non-monotonic average fitness trajectories. B) Denoting δf as the region of fitness values below the steady-state average fitness for which the mean selection coefficient is negative, in the limit of large N δf asymptotes to 0.5 for the dB^p star graph, whereas the gap decays to 0 for the complete graph. Parameters: $N = 100$, $f_{\min} = 0.1$, $f_{\max} = 10$, $\rho(f', f) = \frac{1}{f_{\max} - f_{\min}}$.

m_I, whereas the probability that an individual from the central deme migrates to leaf deme is m_O. The parameter $\alpha = m_I/m_O$ quantifies the migration asymmetry. The probability that the mutant type with relative fitness r takes over the entire star network-structured metapopulation given that the population is initialised with central deme being the mutant deme and leaf demes being the wild-type demes is given by,

$$\Phi_{\bigstar}^{\bullet} = \frac{1-\gamma^2}{1+\alpha\gamma-\gamma(\alpha+\gamma)\left(\frac{\gamma(1+\alpha\gamma)}{\alpha+\gamma}\right)^d}, \tag{61}$$

where

$$\alpha = \frac{m_I}{m_O} \quad \text{and} \quad \gamma = \frac{\Phi_{C_W}}{\Phi_{C,M}} \tag{62}$$

with

$$\Phi_{C,M} = \frac{1-\frac{1}{r}}{1-\frac{1}{r^N}} \quad \text{and} \quad \Phi_{C,W} = \frac{1-r}{1-r^N}. \tag{63}$$

Assuming that the central deme is initialised as the mutant deme, for smaller values of α, the star metapopulation acts as an amplifier of fixation. Conversely, for larger values of α, it functions as a suppressor of fixation, see Fig. 8 (adapted from ref. [19]).

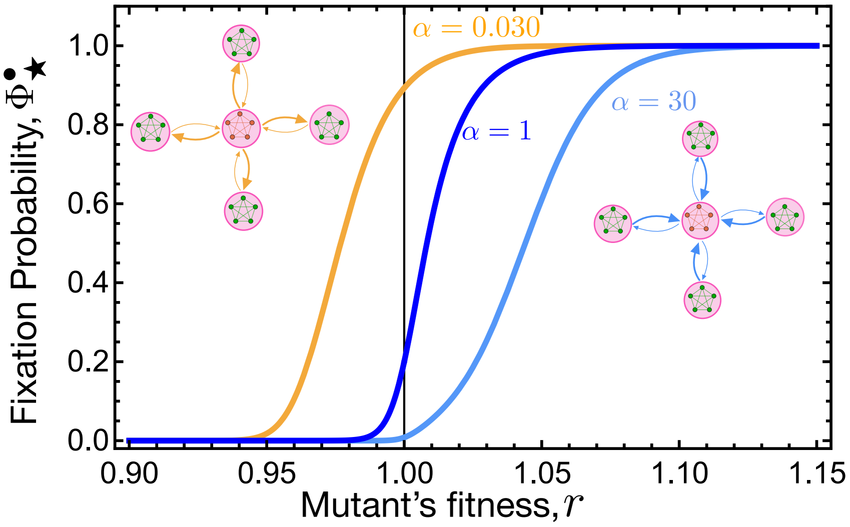

FIG. 8. **Amplifier and suppressor of fixation in meta-star.** The probability that a mutant fixates in a star-structured metapopulation is shown given that the population is initialised with the central deme being the mutant deme. The probability that an individual from the central migrates to a leaf deme is m_O, and the probability of migration for an individual migrating from a leaf node to the central node is m_I. Parameter $\alpha = m_I/m_O$ quantifies the migration asymmetry. According to the generalized circulation theorem in ref. [19], for $\alpha = 1$, the meta-star's fixation probability is the same as in the well-mixed population when started with N mutant individuals. Changing α from 1 gives two different fixation probability profiles: the amplifier of fixation (for lower α) and the suppressor of fixation (for higher α). We have encountered these profiles is the main text for one node one individual star under Bd^o and dB^p. Parameters: size of each deme, $N = 80$ and the total number of demes $D = d + 1 = 5$.

[1] Broom, M. & Rychtář, J. An analysis of the fixation probability of a mutant on special classes of non-directed graphs. *Proceedings of the Royal Society A* **464**, 2609–2627 (2008).
[2] Hadjichrysanthou, C., Broom, M. & Rychtář, J. Evolutionary games on star graphs under various updating rules. *Dynamic Games and Applications* **1**, 386–407 (2011).
[3] Tkadlec, J., Pavlogiannis, A., Chatterjee, K. & Nowak, M. A. Limits on amplifiers of natural selection under death-birth updating. *PLoS computational biology* **16**, e1007494 (2020).
[4] Allen, B. *et al.* Transient amplifiers of selection and reducers of fixation for death-birth updating on graphs. *PLoS computational biology* **16**, e1007529 (2020).
[5] Hindersin, L., Moeller, M., Traulsen, A. & Bauer, B. Exact numerical calculation of fixation probability and time on graphs. *BioSystems* **150**, 87–91 (2016).
[6] Grinstead, C. M. & Snell, J. L. *Introduction to probability* (American Mathematical Soc., Providence, RI, 2012).
[7] Hindersin, L. & Traulsen, A. Counterintuitive properties of the fixation time in network-structured populations. *Journal of The Royal Society Interface* **11**, 20140606 (2014).
[8] Kaveh, K., Komarova, N. L. & Kohandel, M. The duality of spatial death-birth and birth-death processes and limitations of the isothermal theorem. *Royal Society Open Science* **2**

(2015).

[9] Lieberman, E., Hauert, C. & Nowak, M. A. Evolutionary dynamics on graphs. *Nature* **433**, 312–316 (2005).

[10] Nowak, M. A. *Evolutionary dynamics: Exploring the equations of life* (Harvard University Press, 2006).

[11] Hindersin, L. & Traulsen, A. Most undirected random graphs are amplifiers of selection for Birth-death dynamics, but suppressors of selection for death-Birth dynamics. *PLoS Computational Biology* **11**, e1004437 (2015).

[12] Manhart, M., Haldane, A. & Morozov, A. V. A universal scaling law determines time reversibility and steady state of substitutions under selection. *Theoretical population biology* **82**, 66–76 (2012).

[13] Kelly, F. P. *Reversibility and stochastic networks* (Cambridge University Press, 2011).

[14] Otto, S. P. & Day, T. *A Biologist's Guide to Mathematical Modeling in Ecology and Evolution* (Princeton University Press, Princeton, New Jersey, 2007).

[15] Sharma, N. & Traulsen, A. Suppressors of fixation can increase average fitness beyond amplifiers of selection. *Proceedings of the National Academy of Sciences* **119**, e2205424119 (2022).

[16] Frean, M., Rainey, P. & Traulsen, A. The effect of population structure on the rate of evolution. *Proceedings of the Royal Society B* **280**, 20130211 (2013).

[17] Park, S.-C., Simon, D. & Krug, J. The speed of evolution in large asexual populations. *Journal of Statistical Physics* **138**, 381–410 (2010).

[18] McCandlish, D. M., Epstein, C. L. & Plotkin, J. B. The inevitability of unconditionally deleterious substitutions during adaptation. *Evolution* **68**, 1351–1364 (2014).

[19] Marrec, L., Lamberti, I. & Bitbol, A.-F. Toward a universal model for spatially structured populations. *Physical Review Letters* **127**, 218102 (2021).
